# Correction of Local Brain Temperature after Severe Brain Injury Using Hypothermia and Medical Microwave Radiometry (MWR) as Companion Diagnostics

**DOI:** 10.3390/diagnostics13061159

**Published:** 2023-03-18

**Authors:** Oleg A. Shevelev, Marina V. Petrova, Elias M. Mengistu, Mikhail Y. Yuriev, Inna Z. Kostenkova, Sergey G. Vesnin, Michael M. Kanarskii, Maria A. Zhdanova, Igor Goryanin

**Affiliations:** 1 Federal Research and Clinical Centre for Resuscitation and Rehabilitology, 107031 Moscow, Russia; 2 Department of Anaesthesiology and Intensive Care, Department of General Pathology and Pathological Physiology, Institute of Medicine, Peoples’ Friendship University of Russia, 117198 Moscow, Russia; 3 Medical Microwave Radiometry (MMWR) LTD, Edinburgh EH10 5LZ, UK; 4 School of Informatics, University of Edinburgh, Edinburgh EH8 9YL, UK; 5 Biological Systems Unit, Okinawa Institute Science and Technology, Onna 904-0495, Japan

**Keywords:** medical microwave radiometry (MWR), cerebral cortex, vegetative state (VS), Coma Recovery Scale-Revised (CRS-R), minimally conscious state (MCS), hypothermia, temperature heterogeneity, neuroprotection, stress proteins, severe brain injury

## Abstract

The temperature of the brain can reflect the activity of its different regions, allowing us to evaluate the connections between them. A study involving 111 patients in a vegetative state or minimally conscious state used microwave radiometry to measure their cortical temperature. The patients were divided into a main group receiving a 10-day selective craniocerebral hypothermia (SCCH) procedure, and a control group receiving basic therapy and rehabilitation. The main group showed a significant improvement in consciousness level as measured by CRS-R assessment on day 14 compared to the control group. Temperature heterogeneity increased in patients who received SCCH, while remaining stable in the control group. The use of microwave radiometry to assess rehabilitation effectiveness and the inclusion of SCCH in rehabilitation programs appears to be a promising approach.

## 1. Introduction

After emerging from a coma, patients who have experienced severe brain damage often pass into a state of chronic disorder of consciousness (DOC) for an indefinite term, including a vegetative state (VS) and minimally conscious state (MCS) [1,2,3]. The increase in the number of these patients induces significant social and economic problems, complicated by a lack of detailed understanding regarding diagnosis, prediction of outcomes, and determination of the principles of therapy and rehabilitation.

Neurological examination is of primary importance when assessing the condition of patients in VS and MCS. Thus, the use of the Coma Recovery Scale-Revised (CRS-R) enables assessment of the current state of the auditory, visual, motor, speech, and communicative functions and the level of wakefulness of patients, which allows practitioners to identify and register the signs of consciousness and to distinguish VS from MCS [4,5].

The main therapeutic strategy in patients with DOC mainly consists of maintaining vital organ functions, preventing infectious complications, and optimizing nutritional support [6]. The priorities in choosing rehabilitation methods are determined not by the proven effectiveness of a particular strategy, but by their availability in a particular healthcare center, and are often based on assumptions about their possible positive impact on processes that improve brain function [7]. There is no sufficiently deep understanding of the processes of the revival of consciousness after emerging from a coma.

Technological development of an objective instrumental assessment of the rehabilitation dynamics and new approaches to methods aimed to increase the level of consciousness is an urgent problem in the previously described extremely severe patients [8,9].

In this study, we focused on the horizons of implementing a new objective diagnostic methodology that would allow us to understand one of the assumed key pathophysiological mechanisms of impaired consciousness, as well as to define the cause-and-effect linkage within this process and the possibility of subsequent correction of the above-described vicious circle. The adaptation of new strategies could be a valuable supplement to the diagnostic work-up for researchers and clinicians who have to deal with patients after severe brain injury, and enhance the effectiveness of the ongoing rehabilitation program, which in turn will decrease mortality among patients with DOC.

One of the promising research methods for the brain’s functional state is noninvasive passive microwave radiometry (MWR). The basic principle of this method is based on registering the power of tissue’s electromagnetic radiation (EMR) in a microwave range of 1–7 GHz [10]. The EMR power is proportional to the intensity of the metabolism, which allows actual values of the tissue temperature to be obtained at a depth of 4–7 cm from the skin surface. Thus, it becomes possible to measure the temperature of the cerebral cortex. The accuracy of the method is ±0.2 °C, which is demonstrated in comparison with the results of a thermal sensor implanted directly into the brain [11]. In the standard version of the procedure, an antenna is installed on the surface of the scalp for locating the EMR (Figure 1), and the temperature in 18 standard projections of the cortex of the left and right hemispheres (nine symmetrical areas on the right and left) is sequentially measured (Figure 2). This method was previously used by our team in a study of temperature heterogeneity in healthy and damaged brains [12].

Thus, it was demonstrated that in healthy individuals (*n* = 120) the average temperature of the left and right hemispheres varied in the range of 36.74 ± 0.37 °C–36.64 ± 0.32 °C, and the highest possible temperature difference registered between relatively “cold” and “heated” areas of the cerebral cortex did not exceed 2 °C (ΔT), averaging 1.4 ± 0.25 °C. In the acute period of the formation of ischemic cerebral infarction, regardless of the area of localization of the lesion, the average temperature of the right and left hemispheres in patients increased up to 37.94 ± 0.28 °C–38.0 ± 0.45 °C. At the same time, cerebral hyperthermia develops at a normal basal temperature in 32% of patients, meaning its occurrence is hidden. The values of ΔT in patients with extensive areas of ischemic lesion can exceed 4 °C, due to the formation of hyperthermia foci reaching 40–41 °C, and averaging 2.4 ± 0.26 °C.

Investigation of cerebral cortex temperature disruption patterns in patients in VS and in MCS, which developed due to various causes, showed that the average temperature of the cerebral cortex of both hemispheres is in the range of 36.98 ± 0.18 °C (*n* = 69), while ΔT equals 1.2 ± 0.26 °C. It is important to note that the measurements were carried between 12.00–18.00 h, which corresponds to the period of daily acrophase of brain temperature [13].

A correlation analysis demonstrated that healthy participants were characterized by the presence of positive medium strength bonds between the temperature values of nine symmetrical regions of the left and right hemispheres. The correlation coefficients (K, Spearman rank correlation coefficient) ranged from 0.504 to 0.747. On the first day of the ischemic cerebral infarction, K varied from −0.370 to 0.848, demonstrating the development of sufficient temperature heterogeneity of the cerebral cortex. Strong positive correlations between temperature values in the symmetrical regions of the left and right hemispheres were typical for patients with VS and MCS. The K-coefficients ranged from 0.937 to 0.971, reflecting the similarity of the temperature distribution in the cerebral cortex and a decrease in temperature heterogeneity.

These results demonstrated that moderate temperature heterogeneity of the cerebral cortex is typical for healthy individuals, while heterogeneity is significantly increased in patients in the acute period of ischemic incidents, compared to patients with DOC, in which it is reduced.

The brain temperature is an integral marker of the functional activity of its regions, which enables assessment of the nature of the connections between them, with the assumption that temperature heterogeneity reflects functional heterogeneity. This approach allows us to consider indicators of the level of brain temperature heterogeneity from the standpoint of the theory of functional systems [14,15,16].

High temperature heterogeneity of the brain in patients with schizophrenia is typical for the pharmaco-resistant forms of the disease, and remission is accompanied by a reduction of temperature heterogeneity [17]. The detection of such dynamics allows us to assume, that, by using methods of reducing high or increasing low functional (temperature) heterogeneity, it is possible to achieve a positive therapeutic effect.

## 2. Correction of Brain Temperature Balance Disruption in Patients with Severe Brain Injuries

Neurogenic fever and cerebral hyperthermia are not only the markers of acutely developing brain lesions, but also an important link in pathogenesis that worsens the course and outcomes of the disease, which can be stopped by using temperature-lowering technologies [18].

The high neuroprotective capabilities of hypothermia associated with metabolic suppression and the genetic cell response to reduced temperatures make hypothermia attractive for clinical use in the acute period of brain injuries. Current therapeutic hypothermia technologies mostly use general cooling of the patient, decreasing the body temperature to 32–33 °C, which is accompanied by various side effects and complications [19].

Meanwhile, selective hypothermia of the cerebral cortex, achieved by selective craniocerebral cooling (selective craniocerebral hypothermia—SCCH; Figure 3 and Figure 4), allows for a decrease in the temperature of the brain surface at the depth of the local tissues, which is necessary for the suppression of metabolism and expression of cytoprotective genes. By using SCCH, only the surface temperature of the brain is lowered, insignificantly affecting the temperature of the basal structures and body temperature [20]. The use of SCCH in the development of neuroprotection mechanisms has been demonstrated earlier in experimental and in clinical trials, including during the acute period of ischemic stroke [21].

The SCCH methodology involves a cryoapplicator helmet that utilizes channels to circulate coolant and remove surface heat from the scalp. This maintains the scalp temperature at between 3–7 °C, due to the close contact between the helmet and the head surface. The device contains control feedback sensors, including one on the inner surface of the helmet and two supplementary sensors for measuring tympanic and axillary temperature, which helps to maintain the set temperature. After the cooling procedure is complete, the helmet is removed, and the patient undergoes a rapid spontaneous rewarming of the cerebral cortex.

This technique has several advantages, including its ease of use and the possibility of inducing cerebral hypothermia in awake patients, comatose patients, and even healthy individuals. Additionally, local cooling helps to reduce the cold load on the body, which helps to prevent complications that are often associated with general therapeutic hypothermia, such as shivering, muscle tremors, and the need for sedation. 

The therapeutic effects of SCCH on metabolism and gene expression and wider systems biology in the brain cells have been discussed and new directions for patient rehabilitation have been proposed [22,23].

The utility of SCCH in patients with DOC, after severe cerebral injuries, does not seem obvious. The main destructive events have already happened, and the use of hypothermia, especially along with general cooling technologies, bears risks for patients. At the same time, a characteristic feature of the brain temperature balance in this category of patients is a significant decrease in temperature heterogeneity of the cerebral cortex, as well brain circadian rhythm disruption [24]. We have suggested that selective hypothermia of the cerebral cortex, achieved by using SCCH, can be used to increase temperature heterogeneity, providing modification of the cooling procedure.

In the acute period of ischemia, the SCCH procedure has the objectives of lowering the temperature of the cerebral cortex by 4–6 °C and correcting temperature heterogeneity. It was noted that, after a 16–24-h SCCH session, smooth spontaneous rewarming should be used, which strengthens the therapeutic effect. The warming is applied only to the cerebral cortex since the basal temperature does not decrease below the level of very mild hypothermia. The smooth rewarming at the end of the session involves a gradual (with two- or three-hour intervals) increase of the brain temperature from 3–5 °C to 10–12 °C, then to 15–17 °C. This procedure takes about 5–6 h.

The blood flow in the cooled tissues decreases during the SCCH procedure, while smooth rewarming prevents rapid resumption of blood flow. In turn, rapid spontaneous rewarming (for example, when removing the helmet from the patient’s head), leads to a rapid restoration of the blood flow, leading to a reperfusion effect [25].

Presumably, short procedures with rapid spontaneous warming of the cerebral cortex might induce fluctuations in vascular reactions and might affect the level of temperature heterogeneity of the cerebral cortex. The duration of the procedure should allow reduction of the temperature of the cortex by 1.5–2 °C. It is believed that, under these conditions, neuroprotective processes might be initiated.

Since the use of SCCH was found to be safe and did not create any complications or side effects in critical patients, we developed a method of craniocerebral cooling for patients with DOC. The methodology includes a course (10–12 sessions) of short SCCH procedures (120 min), during which the temperature of the scalp is decreased to 3–7 °C and the cerebral cortex by at least 1.5–2 °C. The procedure ends with a quick spontaneous rewarming.

This study aims to define the features of the brain temperature balance of patients in VS and MCS, developed after severe cerebral injuries (ischemic and hemorrhagic stroke, and cerebral lesions), as well as to assess the effects of SCCH on cerebral cortex temperature heterogeneity and processes of restoration of consciousness.

## 3. Materials and Methods

A total of 111 patients with DOC were included in the study. Inclusion criteria were as follows: patients with DOC, developed after severe focal brain damage (including consequences of ischemic and hemorrhagic strokes or severe traumatic brain injury), not earlier than 30 days after cerebral injuries. Exclusion criteria were as follows: anoxic brain damage (due to cardiac arrest or asphyxia) with extensive diffuse damage of the cerebral cortex, sepsis, cardiac arrhythmias, initial hypothermia (body temperature below 35.5 °C), or terminal stages of the disease.

Patients were randomized into two groups. The main group (*n* = 60 patients) included two subgroups. The first subgroup of the main group (O1) included patients in VS (*n* = 39): women (W)—15 (mean age 36.7 ± 4.4), men (M)—24 (mean age 43.3 ± 3.4). The second subgroup of the main group (O2) included patients in MCS-minus (*n* = 21): W—7 (mean age 44.6 ± 7.7), M—14 (mean age 47.5 ± 3.2). The comparison group (*n* = 51 patients) also included two subgroups. The first subgroup of the control group (C1) included patients in VS (*n* = 32): W—20 (mean age 46.9 ± 3.2), M—12 (mean age 44.1 ± 4.1); the second (C2) included patients in MCS-minus (*n* = 19): W—10 (mean age 56.1 ± 3.5), M—9 (mean age 49.2 ± 3.0).

In all subgroups, the results were registered on the first and the fourteenth day. The mortality rate was compared on the thirtieth day of follow-up.

Patients in the main group underwent 10 sessions of SCCH lasting 120 min, during fourteen days of follow-up. Patients in the control group did not undergo SCCH. In both groups, all patients received standard neurotropic therapy and underwent standard rehabilitation procedures.

A device for craniocerebral hypothermia, ATG-01, (Russia Federation) was used (Figure 3). The surface of the craniocerebral region was cooled using a cryoapplicator helmet that maintained the scalp temperature at 3–7 °C (Figure 4). The cooling procedure was completed by removing the helmet, after which the patients underwent rapid spontaneous warming of the cerebral cortex.

The MWR of the cerebral cortex was performed using an RTM-01-RES device (Figure 5). The internal temperature was registered in nine areas of the cerebral cortex projections of the left and right hemispheres (Figure 6), before the first procedure and by the end of the tenth procedure. The temperature values in the projections of the frontal cortex and the axillary cavity were registered before the session, every 30 min until the end of the procedure, and 30 min after termination. The procedure was carried out under standard conditions for a time period of 12–18 h in the intensive care unit, at a 25–27 °C room temperature and 75–80% humidity.

The level of consciousness was assessed according to the Coma Recovery Scale-Revised (CRS-R, 2004) with an assessment of the severity of functions in the following scores: hearing ability, visual function, mobility and speech functions, communication, and the level of wakefulness. The analysis included CRS-R scale data obtained in the main group before the first procedure and on the fourteenth day after the tenth SCCH procedure. In the control group, the data (according to the CRS-R scale) were evaluated on the first day and the fourteenth day.

Statistical analysis was performed using the SPSS Statistics 21.0 application package. The differences were considered significant at *p* ≤ 0.05.

## 4. Results

It was found that the average temperature before the first SCHH procedure in the projection area of the frontal lobes of the left (LH) and right hemispheres (RH) in patients of both groups in VS and MCS-minus did not statistically differ (36.4 ± 0.11 °C and 36.4 ± 0.09 °C, respectively). Correlation analysis revealed the presence of strong positive correlations between symmetrical areas of the cerebral cortex (r = 0.86–0.92), which indicated a low level of temperature heterogeneity of the cerebral cortex. The axial temperature was 36.4 ± 0.09 °C.

After 30 min of SCCH, the temperature of the LH and the RH started to reduce, by the ninetieth minute it reached 33.9 ± 0.38 °C and 33.5 ± 0.53 °C, respectively, and by the end of the procedure it dropped by 2.4–3.1 °C. After removing the cooling helmet from the patient’s head, the temperature in the LH and RH returned to the initial values within an hour. During the entire cooling procedure, the axial temperature did not change.

Analysis of the functions on the first day of the study in patients subgroup O1 of the main group (VS, *n* = 39), according to the CRS-R scale, revealed that the total assessment of the level of consciousness equalled 4.5 ± 0.33, and in patients of the control group in subgroup C1 (VS, *n* = 32) it was 4.3 ± 0.37. On the fourteenth day of the study, after the tenth SCCH procedure in the O1 subgroup, the score reached 8.7 ± 0.91 (*p* < 0.001) and, in the O1 subgroup, it was 6.8 ± 0.49 (*p* < 0.001). In the O1 subgroup, hearing ability, visual function and speech functions, communication and the level of wakefulness increased very significantly (*p* = 0.00083). The motor function increased slightly less (*p* < 0.005). In control subgroup C1, only hearing ability and visual function increased very significantly (*p* < 0.001), motor, speech and communicative functions increased less significantly (*p* < 0.005), and the level of wakefulness remained the same.

The averaged data indicate that patients in VS who received the SCCH procedure reached the MCS-minus level, whereas, in the C1 subgroup, the changes were fewer.

While reflecting the general trend, the average values do not reflect the heterogeneity of the results. Thus, in the O1 subgroup, the best results (CRS-R > 16 points) were obtained in six patients (15.4%); in three patients, CRS-R values reached 16–19 points (MCS-plus), and in three more patients they reached 20–21 points, indicating an approximation to full consciousness. In the control group, C1, the best results (CRC-R > 11–13 points) were achieved in five patients (15.6%), and these corresponded to the MCS-minus level.

According to the CRS-R scale, on the first day, the O2 subgroup patients (*n* = 21) scored a total of 11.3 ± 1.00 points, and the C2 subgroup patients (*n* = 19) scored 9.1 ± 0.57 points. On the fourteenth day of the study, after the course of SCCH in the O2 subgroup, the values increased up to 18.2 ± 0.70 points (*p* < 0.001), speech function increased in patients in the C2 subgroup (*p* < 0.05), and the total score increased, although not significantly, to 10.1 ± 0.86 (*p* > 0.1).

In the O2 patients who underwent a course of SCCH, the best results (CRS-R > 16, MCS-plus) were obtained in eight patients (38.1%), and in five patients in this group, CRS-R values reached 20–23 points, indicating a significant improvement of consciousness. In the control C2 group, in four patients (21%), a level of 12–16 points was reached on the fourteenth day, which corresponded to the MCS-plus level.

Changes in functions according to the CRS-R scale in patients in the main groups and control groups are presented in Table 1 and Table 2.

On the fourteenth day, the correlation analysis revealed an increase in the temperature heterogeneity of the cerebral cortex in patients in VS and MCS, in comparison with the data obtained before the course of hypothermia. There was a wide variety in correlation coefficients (r = 0.36–0.87), which indicated an increase in the level of temperature heterogeneity. The correlation coefficients did not change significantly in the control group of patients (r = 0.83–0.86).

The analysis of the mortality rate carried out after 30 days revealed that six patients of the O1 subgroup (15.4%) died. In the O2 subgroup, all of the patients were alive. In the control group, seven patients (21.9%) died in subgroup C1, while four patients (21.1%) died in subgroup C2. Six patients (10%) died in the main groups, and 11 patients (21.6%) died in the control groups. The leading causes of death in both groups were sepsis, thromboembolic complications, and multiple organ failure. There were no complications or side effects specific to hypothermia reported.

## 5. Discussion

In this pilot study, we tested the hypothesis that the level of temperature heterogeneity of the cerebral cortex may reflect the nature of the disruption of the functional connections between the cortical regions.

Undoubtedly one of the limitations of our study is the moderate sample size; however, considering the high mortality rate in such patients, it is challenging to recruit a larger number of individuals and this could take a long time. Despite this, we are continuing to collect data but, at this intermediate stage, our main aim of the study was to show the possibility of using completely new and safe methods to facilitate diagnosis and increase the rehabilitation potential in such vulnerable patients. The obtained results demonstrate the potential benefit of implementing MWR and SCCH as effective auxiliary techniques in the treatment and rehabilitation of patients with DOC.

The decrease of heterogeneity in the acute period of ischemic stroke [26] and its increase in patients with DOC in this study were accompanied by an improvement in clinical status and consciousness level, to a certain extent confirming this hypothesis.

The mechanisms of positive changes in patients under the influence of hypothermic effects on the brain are associated with well-known effects [27]. They include the following metabolic conditioned reactions that develop with a decrease in cerebral temperature: restriction of oxygen and substrate consumption, inhibition of excitotoxicity reactions and receptor-mediated interactions of signaling molecules, restriction of the development of oedema and inflammatory response, apoptosis, and proliferation [28].

In addition, a small range of temperature variation (1–3 °C) becomes sufficient for the expression of genes encoding a wide range of different stress-protective proteins, including cold shock proteins (CSPs) and heat shock proteins (HSPs) [29,30]. An increase in temperature reduces CSPs production, and warming provokes an increase in HSPs production, even at low temperatures. CSPs and HSPs are generally referred to as stress proteins with a high potential for neuroprotection [31,32].

The representatives of CSPs, RBM3 (RNA binding motif 3) and cold-inducible RNA binding protein (CIRBP), provide a reduction of the negative effect of various damaging factors on neurons, with a decrease in temperature of even 1 °C [33,34], and reduce the amount of damage to neurons caused by hypoxia, increase neuronal tolerance to damage, inhibit apoptosis, and contribute to the restoration of the microtubular system of neurons after damage [35,36].

The temperature increase at the cessation of hypothermia provokes an intensive production of HSP70 and HSP90, which are characterized by the most significant cytoprotective effects [37]. During the period of warming from 33 °C to 37 °C, the expression of CHPs is inhibited, while the expression of HSP70 escalates as the temperature increases [38]. It is assumed that warming after cooling activates cellular respiration and the formation of free radicals that provide signals for the expression of the genes involved in the development of stress-protective reactions.

In this study, we demonstrated that even small temperature fluctuations are a significant signal for the expression of the HSPs. This method of induction of hypothermia, allowing fluctuations in the body temperature of animals within the range of 34–35 °C, facilitated an almost four-fold reduction of the brain lesions caused by occlusion of the middle cerebral artery [39]. The authors associate this powerful neuroprotective response with cooling, accompanied by the activation of the HSPs. The protective role of HSP protein in hypothermia in terms of cerebral ischemia has also been demonstrated in other experimental models [40].

Stress proteins that contribute to the development of neuroprotection and activate the processes of neuroregeneration and neuroplasticity include hibernation proteins. These proteins protect animals during torpor and when emerging from it. Torpor development in spontaneously hibernating animals is associated with the fibroblast growth factors. For example, FGF21 is a key inducer of hibernation and has a direct neuroprotective effect [41], reducing the amount of neuronal death induced by glutamate. FGF21 enhances the neuroprotective effect of the CSPs RBM3 protein, contained in the cortical neurons of rats, and is expressed even during very mild hypothermia (35 °C) [42]. FGF21 promotes remyelination [43], increases the integrity of the BBB (blood–brain barrier), and reduces brain oedema, damage volume and neurological deficit after experimental TBI [44]. This reduces the inflammatory response and the size of brain infarction in an ischemic stroke model [45]. It also improves indicators of biochemical markers of brain damage after hypoxic–ischemic injury in adult rats [46].

UCP proteins (uncoupling proteins) play an important role in warming animals when they emerge from torpor; one of these, rUCP1 (thermogenin) [47], stimulates synaptogenesis and neurogenesis, and demonstrates antioxidant properties. Under the influence of UCP, the expression of the brain neurotrophic factor (BDNF) [48] increases. Another representative of the natural hibernation process, irisin, helps to reduce the volume of brain lesions in rats after occlusion of the medial cerebral artery and inhibits apoptosis reactions [49]. The use of irisin in experimental models stabilized the BBB, stimulated the accumulation of BDNF, and reduced the volume of myocardial and lung damage after total ischemia [50,51].

Lowering the brain temperature to 33 °C reduced the volume of damage during occlusion of the middle cerebral artery in rats and was accompanied by an increase in ubiquitin (ubiquitous) synthesis and protein ubiquitination, which may be one of the important mechanisms of neuroprotection [52,53].

It is important that the effects of the gene expression and the accumulation of stress proteins persist for more than one day. The course of daily SCCH sessions, providing a decrease in the temperature of the brain surface by 2.5–3 °C, could cause the accumulation of stress proteins which, hypothetically, could have a positive effect on the processes of improving consciousness. To a certain extent, these assumptions are confirmed by the clinical results obtained in this study.

## 6. Conclusions

The results of this study emphasize the high value of the use of MWR as companion diagnostics to measure the temperature balance of the cerebral cortex, and demonstrated positive effects of the use of SCCH in patients in VS and MCS (Figure 7). It is important to conduct further in-depth studies, with larger sample sizes, and more precise patient selection according to their age, gender, and aetiology of cerebral injuries, and search for biomarkers that are involved in the processes of improving consciousness in patients with DOC. Although our study is limited by a moderate sample size, it is essential to note that mortality rates for this patient category are high, and collecting data from a larger sample might be time-consuming. Our main priority in conducting this study was to demonstrate the potential use of innovative and safe methods as diagnostic tools and devices to correct pathophysiological processes in comatose patients. This approach could significantly enhance the rehabilitation potential of vulnerable patients.

## Figures and Tables

**Figure 1:**
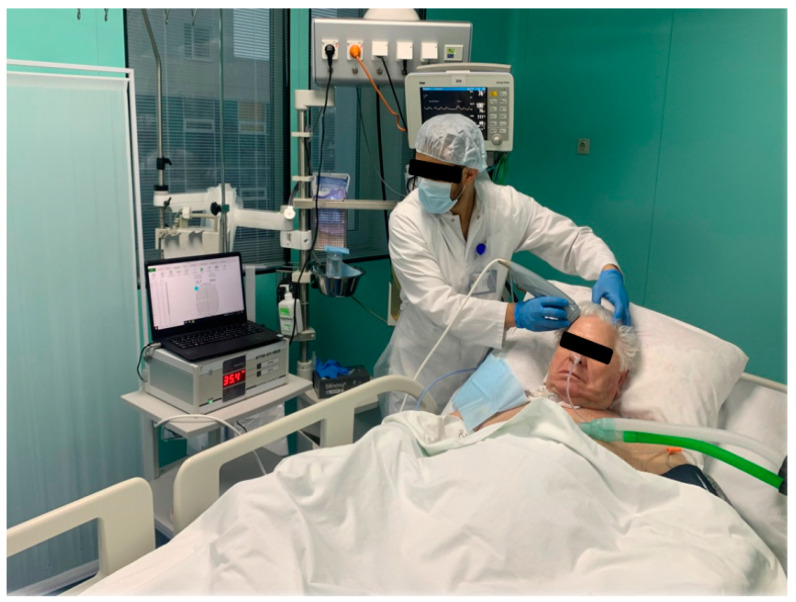
MWR probe in the projection of the right frontal lobe is used to measure the temperature of the cerebral cortex in a patient with DOC.

**Figure 2:**
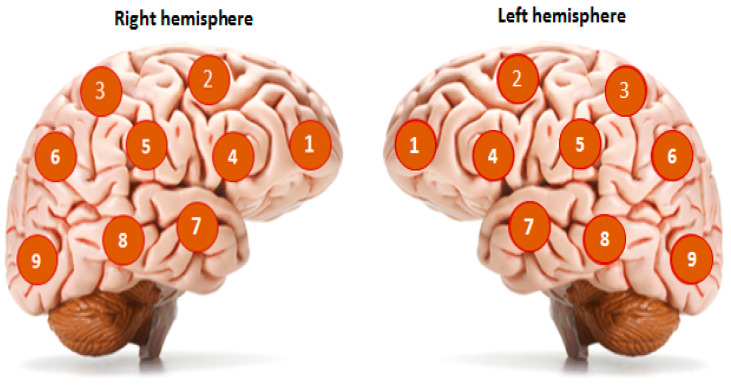
Projection of 18 areas (9 symmetrical areas on the left and right hemispheres) for cerebral cortex temperature measurements using the MWR probe.

**Figure 3:**
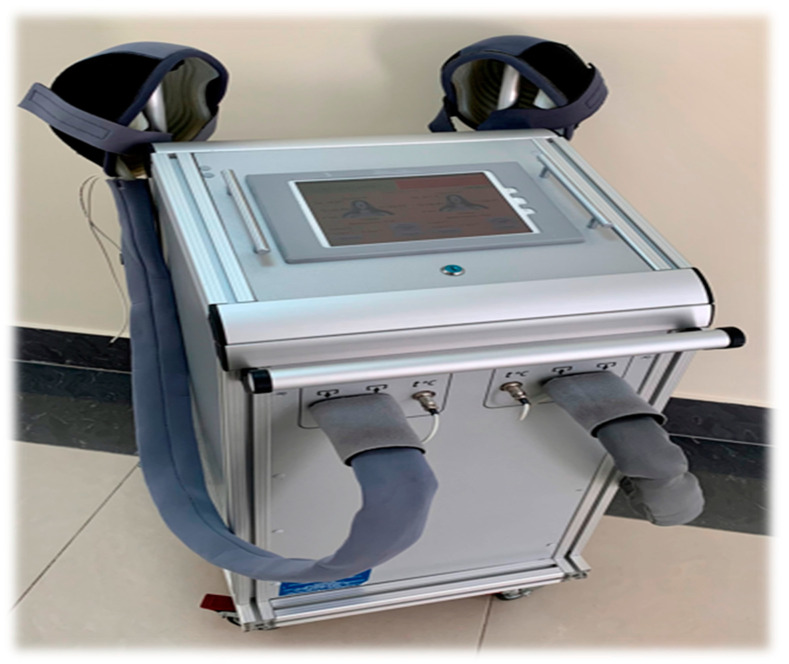
Device for craniocerebral hypothermia (ATG-01).

**Figure 4:**
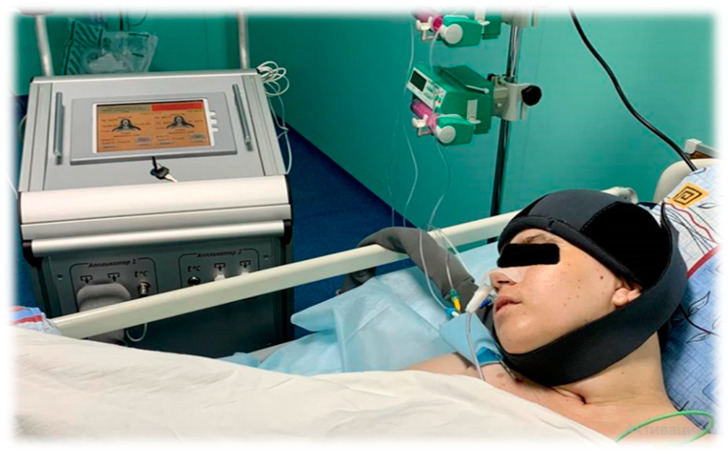
ATG-01 device. Cryoapplicator helmet. The scalp temperature is maintained at 3–7 °C.

**Figure 5:**
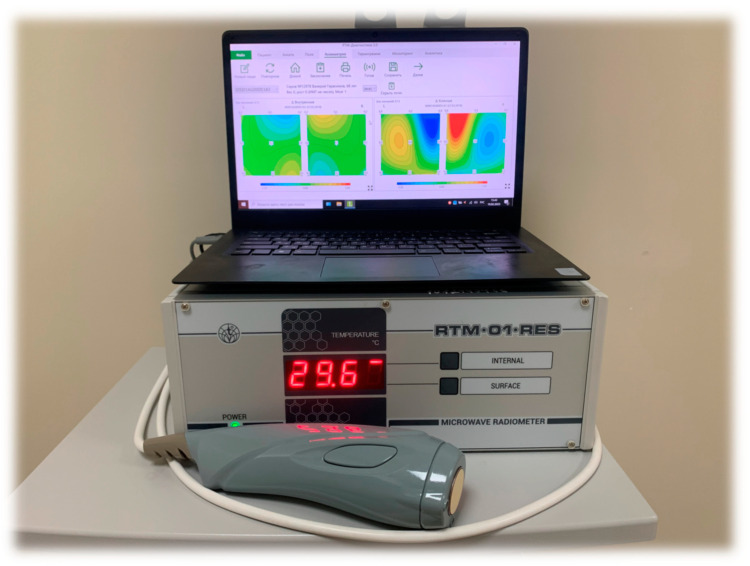
Passive microwave radiometry device (RTM-01-RES).

**Figure 6:**
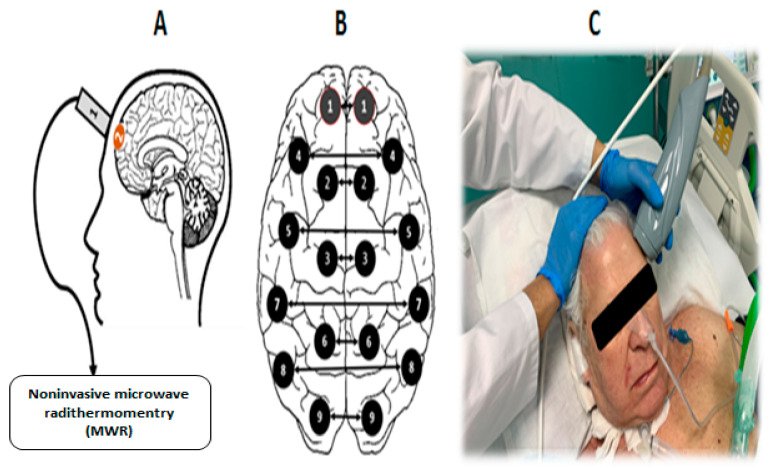
Cerebral cortex temperature registration using MWR. (**A**) →Registration scheme of cerebral cortex temperature in the frontal lobes by using MWR: 1→Antenna location to measure the internal temperature of the cerebral cortex; 2→Area of the internal temperature measurements; (**B**)→Measurement points. Projection of the cerebral cortex (the arrow shows symmetrical areas in patients with DOC); (**C**)→Cerebral cortex internal temperature measurements in the projection of the left frontal lobe using MWR.

**Figure 7:**
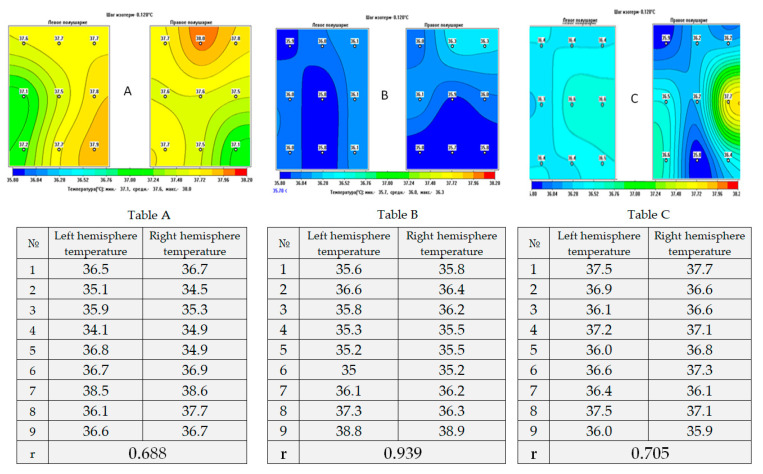
Examples of thermograms obtained by using MWR showing internal brain temperature distribution in the projections of the cerebral cortex in (**A**)—healthy individuals; (**B**)—patient in VS (1st day of the study); (**C**)—patient in VS (after 10 SCCH procedures). Temperature data in the projection of the left (LH) and the right (RH) hemispheres in healthy individuals (Table A), patients in VS on the first day of the study (Table B) and patients in VS after a course of SCCH (Table C) with the calculation of the correlation coefficient (r).

**Table 1:** Changes in functions according to the CRS-R scale in the main groups.

Functions According to the CRS-R Scale	Main Group			
	Subgroup O1		Subgroup O2	
	1st Day	14th Day	1st Day	14th Day
Hearing ability	0.7 ± 0.10	1.5 ± 0.18 ***	2.2 ± 0.23	3.3 ± 0.12 ***
Visual function	0.8 ± 0.11	1.9 ± 0.23 ***	2.6 ± 0.31	4.1 ± 0.22 ***
Mobility	1.3 ± 0.13	2.1 ± 0.24 **	3.1 ± 0.31	4.8 ± 0.19 ***
Speech function	0.4 ± 0.09	0.9 ± 0.13 ***	0.8 ± 0.15	1.8 ± 0.17 ***
Communication	0.1 ± 0.04	0.6 ± 0.11 ***	0.6 ± 0.15	1.5 ± 0.11 ***
Wakefulness	1.3 ± 0.11	1.8 ± 0.14 ***	2.1 ± 0.16	2.8 ± 0.12 ***
Result	4.5 ± 0.33	8.7 ± 0.91 ***	11.3 ± 1.0	18.2 ± 0.70 ***

Note: ** *p* ≤ 0.01, *** *p* ≤ 0.001.

**Table 2:** Changes in functions according to the CRS-R scale in the control groups.

Functions According to the CRS-R Scale	Control Group			
	Subgroup C1		Subgroup C2	
	1st Day	14th Day	1st Day	14th Day
Hearing ability	0.7 ± 0.11	1.3 ± 0.11 **	1.6 ± 0.16	1.5 ± 0.19
Visual function	0.8 ± 0.10	1.3 ± 0.10 ***	1.8 ± 0.16	2.1 ± 0.21
Mobility	1.2 ± 0.15	1.7 ± 0.11 **	2.3 ± 0.18	2.4 ± 0.27
Speech function	0.2 ± 0.07	0.6 ± 0.12 **	0.7 ± 0.15	1.1 ± 0.17 *
Communication	0.2 ± 0.07	0.5 ± 0.12 **	0.9 ± 0.15	1.0 ± 0.20
Wakefulness	1.3 ± 0.12	1.5 ± 0.13	1.8 ± 0.16	2.0 ± 0.13
Result	4.3 ± 0.37	6.8 ± 0.49 ***	9.1 ± 0.57	10.1 ± 0.86

Note: * *p* ≤ 0.05, ** *p* ≤ 0.01, *** *p* ≤ 0.001.

## Data Availability

Not applicable.
